# Epigenetic regulation of L1CAM in endometrial carcinoma: comparison to cancer–testis (CT-X) antigens

**DOI:** 10.1186/1471-2407-13-156

**Published:** 2013-03-26

**Authors:** Uwe Schirmer, Heidi Fiegl, Marco Pfeifer, Alain G Zeimet, Elisabeth Müller-Holzner, Peter K Bode, Verena Tischler, Peter Altevogt

**Affiliations:** 1 Department of Translational Immunology, German Cancer Research Center, D015, Heidelberg, D, 69120, Germany; 2 Department of Gynecology and Obstetrics, Medical University of Innsbruck, Innsbruck, A, 6020, Austria; 3 Institute of Surgical Pathology, University Hospital Zürich, Zürich, Switzerland

## Abstract

**Background:**

L1CAM was originally identified as an adhesion molecule involved in neural development. In many human carcinomas L1CAM is over-expressed and is associated with a bad prognosis. We previously reported that L1CAM was absent in the vast majority of endometrioid endometrial carcinomas (ECs) (type 1) but was strongly expressed in the more aggressive serous and clear-cell ECs (termed type 2). The differential regulation of L1CAM in ECs is not well understood. Recent evidence suggests that it can be regulated by epigenetic mechanisms. Here we investigated the role of DNA-methylation of the L1CAM promoter for expression. We also studied the relationship to cancer testis (CT-X) antigens that co-localize with L1CAM on chromosome Xq28, a region that is often activated in human tumors.

**Methods:**

We used EC cell lines and primary tumor tissues for our analysis. For expression analysis we employed RT-PCR and Western blotting. DNA-Methylation of the L1CAM promoter was determined after bisulfite conversation and DNA sequencing. Tumor tissues were examined by immunohistochemical (IHC) staining.

**Results:**

We demonstrate that the treatment of L1CAM low/negative expressing EC cell lines with 5^′^-Azacytidine (5-AzaC) or knock-down of DNMT1 (DNA methyltransferase 1) as well as the HDAC (histone deacetylase) inhibitor Trichostatin A (TSA) up-regulated L1CAM at the mRNA and protein level. The L1CAM gene has two promoter regions with two distinct CpG islands. We observed that the expression of L1CAM correlated with hypermethylation in promoter 1 and 5-AzaC treatment affected the DNA-methylation pattern in this region. The CT-X antigens NY-ESO-1, MAGE-A3 and MAGE-A4 were also strongly up-regulated by 5-AzaC or knock-down of DNMT1 but did not respond to treatment with TSA. Primary EC tumor tissues showed a variable methylation pattern of the L1CAM promoter. No striking differences in promoter methylation were observed between tumor areas with L1CAM expression and those without expression.

**Conclusions:**

L1CAM expression correlated with methylation of the L1CAM promoter in EC cell lines. In negative cell lines L1CAM expression is up-regulated by epigenetic mechanism. Although genes localized on Xq28 are often re-expressed by human tumors, L1CAM and CT-X antigens show distinct regulation in response to HADC inhibitors and 5-AzaC.

## Background

The L1 cell adhesion molecule (L1CAM) was originally identified as a neural adhesion molecule involved in brain development. Work in the past has shown that L1CAM is also overexpressed in many human tumors [1,2]. It was shown that L1CAM augments cell motility, invasion and metastasis formation [1-3]. Generally, its expression in a variety of tumors is associated with a bad prognosis [4-7].

L1CAM is absent in normal endometrium [8]. In endometrial carcinomas (ECs), expression is absent in most of the indolent endometrioid type EC (type 1 tumor) but present in the more malignant forms of serous-papillary and clear cell carcinoma (type 2 tumors) [8]. In addition, ECs often occur as a mixed-type, i.e. they are composed of a mixture of endometrioid and serous/clear cells components that can be morphologically distinguished. Importantly, the expression of L1CAM is also mixed and L1CAM staining of IHC sections can be used to identify even minor components of serous/clear cell components [8].

The regulation of L1CAM expression at the transcriptional and/or epigenetic level is not well understood. The L1CAM gene is located at chromosome Xq28 and spans about 26 kb with 29 exons, whereof 28 are protein coding exons [9]. The full-length open reading frame consists of 3,825 bp encoding for a 1,275 amino acid polypeptide [9]. During the past years L1CAM was shown to be subject of epigenetic regulation. Kuwajima *et al.* demonstrated that histone deacetylase inhibitors like butyrate and TSA can upregulate both mRNA and protein levels of the cell adhesion molecules Mel-CAM and L1CAM in B16-BL6 melanoma cells [10]. Another report investigated the methylation status at the L1CAM promoter and found an inverse correlation of DNA methylation and protein expression in both colorectal cancer (CRC) cell lines and CRC patients [11]. Treatment with the demethylating agent 5-AzaC induced L1CAM mRNA/protein expression in two L1CAM negative CRC cell lines, whereas levels of two L1CAM positive CRC cell lines did not change [11]. However, these findings have neither been confirmed nor extended to other tumor entities.

On Xq28, L1CAM colocalizes with CT-X antigens such as the MAGE-A family and NY-ESO-1 that are frequently overexpressed in human tumors. A recent study in prostate cancer has identified Xq28 as one of 35 domains in the prostate cancer genome that undergo activation due to long-range epigenetic remodelling [12].

In the present study we wished to clarify i) whether L1CAM expression in ECs involves epigenetic mechanisms in cell lines and primary tumor tissues and ii) whether L1CAM and the CT-X genes, all encoded in the same locus on the X-chromosome, bear some similarity in their epigenetic regulation.

## Methods

### Cell lines and cell culture

The EC cell lines were maintained in DMEM/F12 medium or RPMI-1640 (PAA Laboratories, Pasching, Austria) supplemented with 10% fetal calf serum as described before [8,13,14].

### Chemicals and antibodies

Antibodies to the ectodomain of L1CAM (monoclonal antibody (mAb) L1-11A, a subclone of UJ127.11) and L1-9.3 were described before [15,16]. Antibodies for detection in Western blot were as follows: GAPDH (Santa Cruz Biotechnology, Heidelberg, Germany), Acetyl-H3 (9765, New England Biolabs), MAGE-A4 (WH4103M1, Sigma-Aldrich, Taufkirchen, Germany), MAGE-A3 (NBP1-02506, Novus Biologicals, Littleton, USA) and Ny-ESO-1 (Invitrogen, Eggenstein, Germany). 5-AzaC, TSA and VA were obtained for Sigma-Aldrich and dissolved in serum-free medium or DMSO.

### RNA extraction, reverse transcription and RT-PCR analysis

RNA extraction from cell lines and Reverse transcriptase reaction were described before [14]. Specific primers and probes for L1CAM, MAGE-A4, NY-ESO-1 and β-actin as internal standard were determined with the computer program “Primer Express” (Applied Biosystems, Foster City, CA). To prevent amplification of contaminating genomic DNA, the probe was placed at a junction between two exons. Primers were produced by Sigma-Aldrich. All primers were used in a concentration of 300 μM. The sequences for the primers were as follows:

L1CAM forward 5^′^-ACGAGGGATGGTGTCCACTTCAAA-3^′^, L1CAM reverse 5^′^-TTATTGCTGGCAAAGCAGCGGTAG-3^′^; β-actin forward 5^′^-ACAAGATGAGATTGGCATGGC-3^′^, β-actin reverse 5′-GCCACATTGTGAACTTTGGGG-3^′^; 5^′^-DNMT1 forward AAGAACGGCATCCTGTACGGAGTT-3^′^, DNMT1 reverse 5^′^-TGCTG CCTTTGATGTAGTCGGAGT-3^′^; MAGE-A3 forward 5^′^-AGCAAAGCTTCCAGTTCCTTGCAG-3^′^, MAGE-A3 reverse 5^′^-ACAGTCGCCCTCTCTTGCGATTAT-3^′^; MAGE-A4 forward 5^′^-TAATCCTGCGCGCTATGAGTTCCT-3^′^, MAGE-A4 reverse 5^′^-TGACCACATGCTCCAGGACTTTCA-3^′^; NY-ESO-1 forward 5^′^-AGTTCTACCTCGCCATGCCTTT-3^′^, NY-ESO-1 reverse 5^′^-TCGGATAGTCAGTATGTTGCCGGA-3^′^.

To determine the mRNA expression levels, 10 ng of cDNA was analysed in triplicates. The PCR reactions were performed with the SYBRgreen Master Mix from Applied Biosystems using an ABI 7300 analyser.

### siRNA transfection

24 h before siRNA treatment 1.5 × 10^5^ cells were seeded per 6-well. The transfection was carried out with Interferin (Polyplus, Illkirch, France) following the manufacturer’s protocol. For each well the final siRNA concentration was 10 nM. After the first transfection the cells were incubated for 72 h under standard conditions and then transfected again and analyzed 48 h after the second transfection. siRNA’s used for the knock-down were as follows: siDNMT1 5^′^-AGACCAGGAUGAGAAGAGA-3^′^, siGFP 5^′^-GGCCAGGUCCAGCAGCGCACC-3^′^. All siRNA’s were synthesized by MWG Eurofines (Ebersberg, Germany).

### Treatment of cells and biochemical analysis

Cells were seeded in 6-well plates and treated for 5 days with 5-AzaC or for 24 h with TSA or VA, respectively. After treatment, the cells were lysed for 15 min at 4°C in RIPA lysis buffer (50 mM Tris–HCl, pH 7.5, 150 mM NaCl, 1 mM EDTA, 1 mM PMSF, 1 μg/ml leupeptin, 5 μg/ml aprotinin, 1% NP40, 0.5% deoxycholic acid sodium salt, 0.1% SDS) and sonified. After centrifugation at 10000 × g for 10 min at 4°C, supernatant was collected and protein concentrations were determined with a commercial protein assay (Pierce, BCA protein assay, Thermo Scientific, Waltham, USA). For Western blot analysis, 50 μg of protein per lane was separated on 10 or 12% SDS-polyacrylamide gels under reducing conditions and transferred onto Immobilon membranes (Millipore, Germany). Protein loading was controlled by Ponceau red staining of the membranes. After blocking for one hour in Tris-buffered saline (TBS) supplemented with 5% non-fat milk and 0.1% Tween 20 (Sigma-Aldrich GmbH, Taufkirchen, Germany), membranes were incubated for one hour at room temperature in blocking buffer containing the respective primary antibody. Membranes were washed three times in TBS-Tween and incubated for one hour with horseradish peroxidase conjugated anti-rabbit or anti-mouse secondary antibody. Immunodetection was performed with a chemoluminescence system (ECL, GE Healthcare, Freiburg, Germany). Protein band intensities were defined as the mean of pixels within the area (mean) of the band limited by a preformed rectangular area (area) after subtraction of the background pixels. Quantification was carried out using the “ScionImage” (Scion Corp.) software.

### Patient cohort and immunohistochemistry

Normal testicular tissue of 10 patients (age range 23–75, median 32) who were orchidectomied between 1994–1996 at the University Hospital Zurich was assembled on a tissue microarray. All patients were resected because of primary testicular germ cell tumors or primary funicular or paratesticular neoplasia (myxoid liposarcoma, well differentiated liposarcoma, monophasic synovial sarcoma). The project has been approved by the local ethics committee (Kanton of Zurich reference number StV 25–2008).

Mouse mAb to MAGE-A4 was kindly provided by the Ludwig Institute for Cancer Research and diluted 1:50. Mouse mAb to NY-ESO1 (Zymed Laboratories Inc.) was diluted 1:50. Mab to L1CAM (clone 14.10) was diluted 1:200. Two protocols were applied: First, on a Ventana Benchmark® platform (Ventana Medical Systems, Tucson, AZ, USA). Here the pretreatment with 60 min boiling in pH 8 Tris buffer was followed by incubation with primary mAb (MAGEA4, NY-ESO1) for 60 min at room temperature (RT) and development with the Ultraview-HRP kit, including incubation with respective secondary antibody for 16 min at RT. Second, on a Leica Bond® platform (Vision Biosystems, Melbourne, Australia), the H2 standard pre-treatment with 60 min boiling in pH8 Tris buffer was followed by incubation with primary mAb (L1CAM for 30 min at RT and development with the Refine-DAB Bond kit, including incubation with secondary antibody for 30 min at RT and additional polymer amplification. All primary antibodies were diluted in Tris/BSA and all staining conditions were predetermined. For negative control the primary antibody was omitted. For both systems, hematoxylin counterstains were applied. H&E staining were performed according to standard protocols.

### Microdissection, DNA isolation and methylation analyses

ECs were collected at the Department of Gynecology and Obstetrics, Medical University of Innsbruck. The project has been approved by the local ethics committee (University of Innsbruck ,UN3801; reference number: 282/4.12).

In total, we analyzed 9 endometrioid ECs (8 endometrioid ECs with areas of squamous differentiation), 7 clear cell ECs, 10 papillary serous ECs and 4 normal endometrial tissues. Immunohistochemistry for L1CAM was conducted as described above. DNA from punch biopsies was isolated using the DNeasy Tissue Kit (Qiagen, Hilden, Germany). Not from all tissue samples DNA of high enough quality for further analysis could be recovered. Therefore we restricted our analysis to those tumors where paired samples from L1CAM positive and L1CAM negative areas were available. Genomic DNA from cell lines was isolated using the AllPrep DNA/RNA/protein kit from Qiagen (Hilden, Germany). Bisulfite modification was performed using the EZ DNA Methylation-Gold Kit (Zymo Research, Orange, CA, USA) according to the manufacturer’s instructions. MethyLight analysis was done as described previously [17]. Briefly two sets of primers and probes, designed specifically for bisulfite-converted DNA, have been used: a methylated set for the gene of interest and a reference set, collagen, type II, alpha 1 (*COL2A1*), to normalize for input DNA. Specificity of the reactions for methylated DNA has been confirmed separately using SssI (New England Biolabs)-treated human white blood cell DNA (heavily methylated). The percentage of fully methylated molecules at a specific locus was calculated by dividing the *GENE*:*COL2A1* ratio of a sample by the *GENE*:*COL2A1* ratio of SssI-treated controls and multiplying by 100. Primers and probes for *COL2A1* have been described before [18]. Primers and probes for L1CAM were determined with the assistance of the computer program Primer Express version 2.0.0 (Applied Biosystems, Foster City, CA, USA) to produce a 68-base-pair PCR amplicon (nucleotide positions c4070008-4069940 as defined by GenBank accession number NT_167198.1; -10,671 nucleotides to −10,603 nucleotides upstream from the transcription start site). Genomic DNA not treated with bisulfite (unmodified) was not amplified with the primers (data not shown). Primer sequences were: L1CAM forward 5^′^-AATACTCCCTTAACCTCGACCTAACC-3^′^, L1CAM reverse 5^′^-GGCGTTGCGTGTAGGTGTT-3^′^; L1CAM Taq Man probe 5^′^FAM-TCGACGACGCCGACCAACGAT-3^′^BHQ1. The amplicon was placed in the promoter 1 region [14].

CpG islands in the analyzed genes were identified using a CpG island searcher (http://www.uscnorris.com/cpgislands/cpg.cgi) which screens for CpG islands which meet the criteria and algorithm described by Takai and Jones [19].

For L1CAM-bisulfite sequencing the following primers were used:

PP1 forward 5^′^-TTAGAGAGTTGGAGGAAAATTTG-3^′^, PP1 reverse 5^′^-ACACACACACACAAAACAAAAC-3^′^; PP2 forward 5^′^-GAGTTTTGTTTTGTGTGTGTGTG-3^′^, PP2 reverse 5^′^-CACCCTAACCCCTAATACCAAC-3^′^, PP3 Forward 5^′^-AGTAGTTGAAGGGAGTTTGG-3^′^, PP3 reverse 5^′^-TAAAAAAAACCCAAAACCTC-3^′^. The primers were determined with the assistance of the computer program Methyl Primer Express software v1.0 (Applied Biosystems, Foster City, CA, USA).

PCR reactions were performed in a final volume of 50 μl containing 2 U of HotStarTaq DNA Polymerase (Qiagen, Hilden, Germany), 0.2 μM dNTP mix (Qiagen), 250nM of each primer, 1x buffer and 150 ng of bisulfite modified DNA. The thermal cycling conditions comprised an initial denaturation step at 95°C for 15 min, 35 cycles at 94°C for 1 min, 55°C, 58°C or 54°C respectively (for PP1, PP2 or PP3 respectively) for 45 sec and at 72°C for 1 min, and after the last cycle an incubation step at 72°C for 10 min. For visualization and statistical analysis of the raw bisulfite sequencing data the free BiQ Analyzer tool was used [20].

### Statistical analysis

For the analysis of statistical significance the Student’s *t*-test was used. P-values in the figures are indicated as follows: * < 0.05, ** < 0.01 *** < 0.001.

## Results and discussion

### Epigenetic regulation of L1CAM in EC cell lines

**Figure 1:**
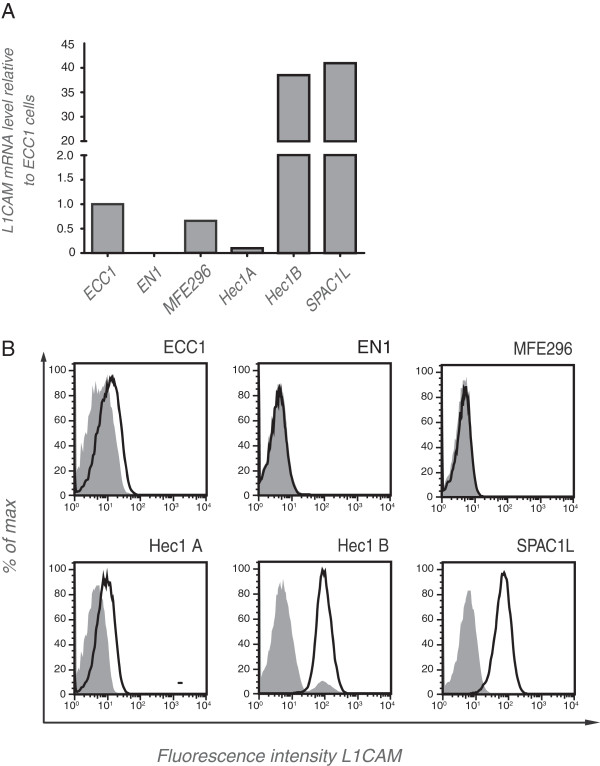
**L1CAM expression in endometrial carcinoma cell lines.** (**A**) mRNA was isolated from the indicated cell lines, transcribed to cDNA and subjected to quantitative RT-PCR analysis. β-actin served as internal standard. (**B**) FACS analysis of selected cell lines. Cells were stained with mAb L1-9.3 (solid line) to the ectodomain of L1CAM followed by PE-conjugated goat anti mouse IgG. For background control (shaded curves) the primary antibody was omitted.

We examined a panel of endometrial carcinoma cell lines (for reference see [13]) for the expression of L1CAM. We identified cell lines with low/negative (ECC1, HEC1A, EN1, MFE 296) or high expression (HEC1B, SPAC1L) at the mRNA level (Figure 1A). FACS analysis of stained cells confirmed the differential expression at the cell surface (Figure 1B).

**Figure 2:**
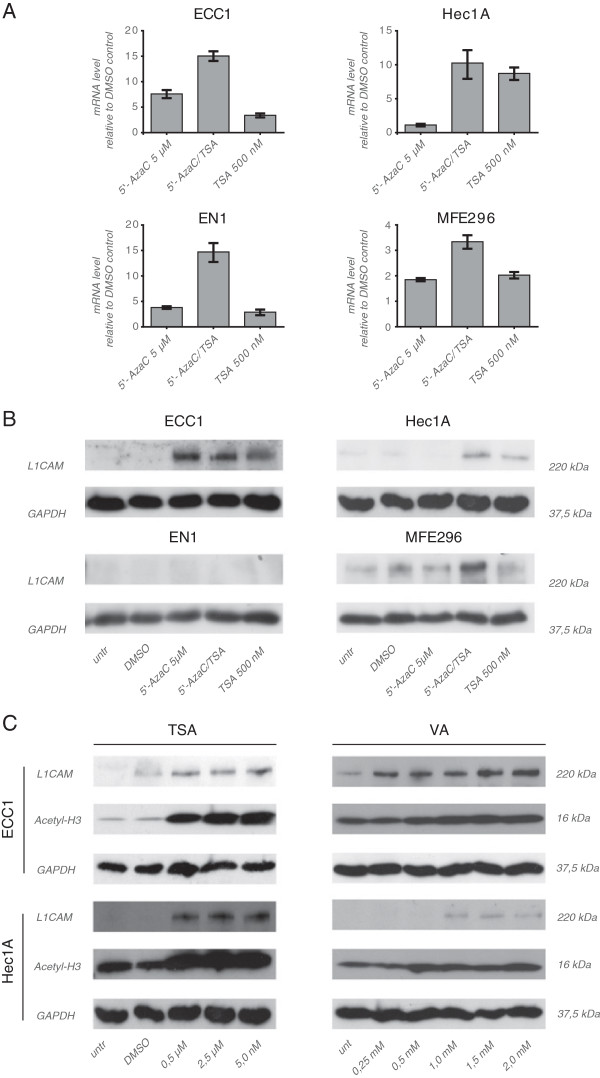
**Regulation of L1CAM expression by epigenetic mechanisms.** (**A**) RT-PCR analysis of cells treated for 5 days with the indicated concentration of 5-AzaC, TSA or both compounds. DMSO was used as a mock control. Cells were lysed and mRNA was isolated and transcribed into cDNA. β-actin served as internal standard. (**B**) Cells were treated as described above and cell lysates were prepared for Western blot analysis. MAb L1-11A was used as a primary antibody followed by peroxidase conjugated Goat anti mouse IgG and ECL detection. (**C**) TSA and VA up-regulate L1CAM expression. Cells were treated and analyzed as described in (**B**).

It was reported before, that treatment of cells with the DNA-demethylating agent 5-AzaC or the broad HDAC inhibitor TSA can lead to L1CAM expression [10,11]. Indeed, a significant induction of L1CAM was observed by RT-PCR in ECC1, HEC1A, EN1 and MFE296 cells treated with both compounds alone or in combination (Figure 2A). Western blot analysis of cell lysates revealed that in ECC1, HEC1A and MFE296 cells these changes were also present at the L1CAM protein level (Figure 2B). In all cases the combination of 5-AzaC and TSA showed the strongest stimulatory effects.

We next tested the effect of the selective HDAC-1,2 inhibitor VA. Indeed, the treatment with TSA or VA up-regulated L1CAM in a dose dependent manner (Figure 2C). Collectively, these results confirmed and extended published data showing that L1CAM can be regulated by epigenetic mechanisms.

### Methylation of the L1CAM promoter in EC cell lines

**Figure 3:**
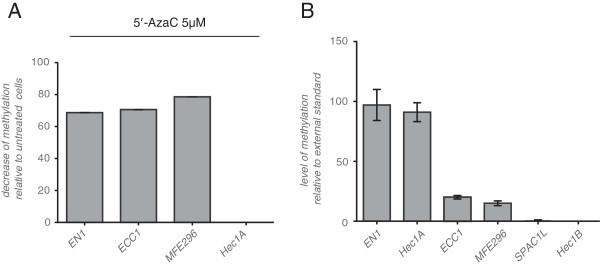
**MethyLight analysis of promoter 1 of the L1CAM promoter region.** (**A**) The indicated cell lines were treated with 5-AzaC or DMSO and the *L1CAM* promoter was subjected to MethyLight PCR. (**B**) MethyLight analysis of the *L1CAM* promoter region in EC cell lines. The analyzed region comprised fragment PP1 (see Figure 4A).

The L1CAM promoter has two transcription start sites, the first in front of the non-translated exon 0 (promoter 1) and the second next to the first coding exon 1 (promoter 2) [14,21,22]. Both sites are active in EC cell lines and are used in a cell-type specific manner [14]. To verify that 5-AzaC treatment changed the methylation status of *L1CAM* promoter, we carried out MethyLight PCR reactions of a region located within promoter 1. In EN1, ECC1 and MFE296 cells a significantly reduced methylation of the L1CAM promoter was achieved by 5-AzaC treatment (Figure 3A). In contrast, in HEC1A cells no changes were observed (Figure 3A). Proliferation control experiments run in parallel suggested that these cells were mostly resistant to treatment (not shown).

The degree of DNA methylation within the L1CAM promoter region selected was quite different between the EC cell lines (Figure 3B). The L1CAM positive lines HEC1B and SPAC1L showed the lowest level of methylation whereas the L1CAM negative cell lines were highly methylated (see below).

**Figure 4:**
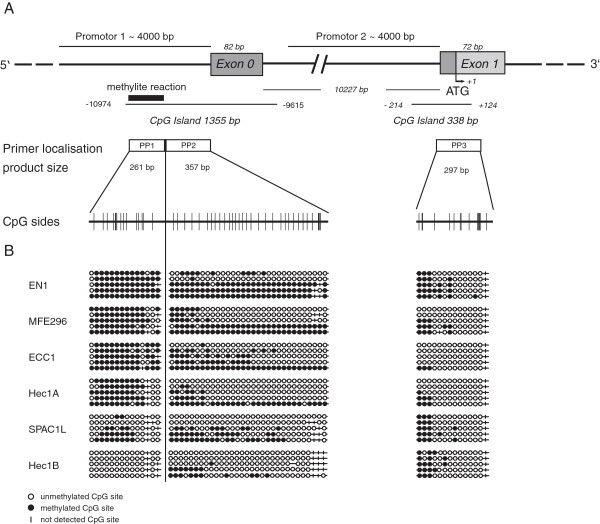
**Methylation of the *L1CAM *promoter in EC cell lines.** (**A**) Schematic illustration of the *L1CAM* promoter region according to the Ensembl database. Exon 1 contains the ATG and the transcription start sites. The upstream sequence of appr. 4100 bp was identified as promoter 2. The non-coding Exon 0 (82 bp) is located appr. 10 kb upstream and is followed by an appr. 4.5 kb promoter region (promoter 1). CpG islands are also indicated. PP1, 2 and 3 indicates fragments that were analysed by sequencing after bisulfite conversion. CpG sides are indicated. (**B**) Schematic illustration of methylation sides in promoter 1 and promoter 2 of the cell lines analysed.

Promoter 1 and promoter 2 of *L1CAM* co-localize with two prominent CpG islands as depicted in Figure 4A. To assess their methylation status, we carried out bisulfite conversion and sequencing of the respective regions. The data are schematically displayed in Figure 4B and statistically summarized in Table 1. Collectively, our results suggested that the level of L1CAM expression is inversely correlated with CpG island 1 methylation. In contrast, the CpG island 2 showed no such correlation. The absence of methylation in CpG islands is typically associated with the activity of genes. It is therefore likely that the binding of transcription factors associated with the regulation of L1CAM in tumors such as β-catenin/TCF-LEF and SLUG [14,22,23] could be facilitated.

**Table 1:** Methylation status of the two CpG islands (CpG1 and CpG2) in the L1CAM promoter of EC cell lines

	**CpG 1**			**CpG 2**		
**Cell line**	**All CpG sites**	**Methylated CpGs**	**%**	**All CpG sites**	**Methylated CpGs**	**%**
EN1	225	136	60.4	70	24	34.3
MFE296	225	129	57.3	70	12	17.1
ECC1	287	116	40.4	70	7	10.0
Hec1A	225	80	35.5	84	3	3.6
SPAC1L	256	69	26.9	84	22	26.2
Hec1B	225	19	8.4	84	19	22,6

### Methylation of the L1CAM promoter in EC tumor tissues

**Figure 5:**
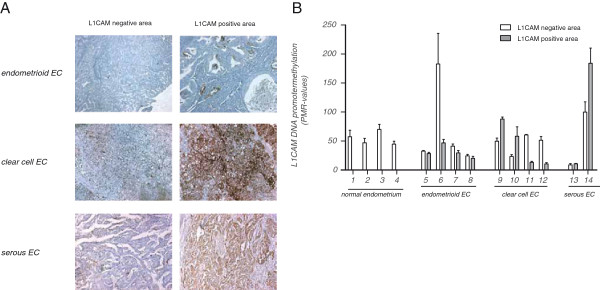
**L1CAM promoter Methylight analysis of EC tumor tissues.** (**A**) Representative staining examples of EC tissues with L1CAM negative and L1CAM positive staining areas. (**B**) Results of the Methylight reaction from paired tumor samples and normal endometrium (numbered from 1 to 14) are shown. DNAs were extracted from punched areas (1 mm diameter) that were selected according to IHC staining. Note that normal endometrium is L1CAM negative.

It is now well known that the methylation patterns in cell lines maintained in long term culture are fraught with potential problems and may diverge from the parental tissue. We therefore extended the MethyLight PCR analysis to primary tumor tissues and extracted DNA from various types of ECs and from normal endometrium tissue that is L1CAM negative. DNAs were extracted from both L1CAM positively or negatively stained tumor areas (see Figure 5A). The results from the Methylight reaction from paired areas of the same tumors are summarized in Figure 5B and show that the L1CAM promoter methylation has a high degree of variability. A tendency for hypermethylation was seen in the L1CAM positive staining areas of some EC tumors but the contrary was noted in other samples (Figure 5B). The differences did not reach statistical significance (not shown).

### Comparison of L1CAM to NY-ESO-1 and MAGE-A3/4

**Figure 6:**
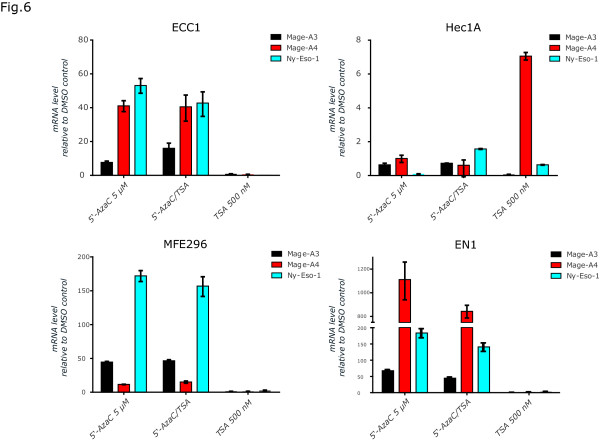
**Analysis of MAGEA and NY-ESO-1 expression.** RT-PCR analysis of cells treated for 5 days with the indicated concentration of 5-AzaC, TSA or both compounds. DMSO was used as a mock control. Cells were lysed and mRNA was isolated and transcribed into cDNA. β-actin served as internal standard.

L1CAM is localized on the X-chromosome in Xq28 in close proximity to the loci for NY-ESO-1 and MAGE-A. To analyse whether the latter genes, in relation to L1CAM, are differentially regulated we compared the effects after treatment of cells with 5-AzaC, TSA or the combination of both compounds. As expected, MAGE-A4, A3 and NY-ESO-1 were up-regulated by 5-AzaC or 5-AzaC/TSA, however, the cell lines differed in their responsiveness. The weakest response to 5-AzaC was seen in HEC1A cells. There were no effects (or a minor effect for HEC1A) of TSA treatment alone (Figure 6). The failure of TSA to up-regulate CT-X genes was confirmed by Western blot analysis (data not shown). These results indicated that in comparison to L1CAM the CT-X antigens are less sensitive to TSA induced regulation but equally sensitive to DNA methylation changes. Moreover, the sensitivity varied depending on the cell lines tested and the CT-X antigen examined.

### DNMT1 knock-down mediates upregulation

**Figure 7:**
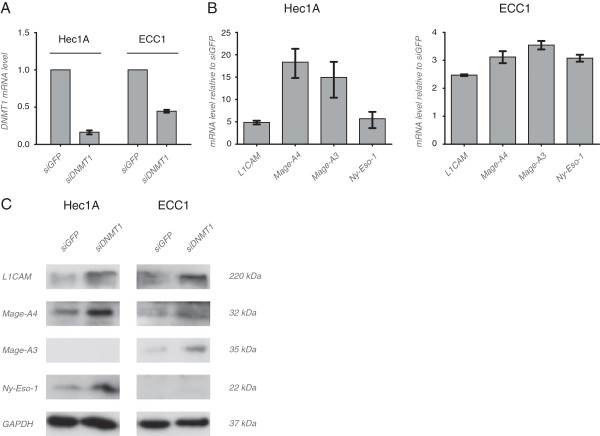
**Analysis of expression after DNMT1 knock-down.** (**A**) DNMT1 was depleted by siRNA in HEC-1A and ECC1 cells and the efficacy of the knock-down was analyzed by RT-PCR. (**B**) Effect of DNMT1 depletion on the expression of the indicated gene products as analyzed by RT-PCR. siGFP was used as control. (**C**) Cells were treated as described above and cell lysates were prepared for Western blot analysis. Primary antibodies to the indicated proteins were used followed by peroxidase conjugated Goat anti mouse IgG and ECL detection. Representative blots from n = 2 experiments are shown.

To further study the regulation of *L1CAM* and *CT-X* genes by DNA-demethylation, we knocked-down the major methyltransferase DNMT1. Significant depletion was achieved in HEC1A and ECC1 cells compared to siGFP controls (Figure 7A). In line with the results obtained with 5-AzaC, the knock-down of DNMT1 upregulated the mRNA of L1CAM, MAGE-A4, MAGE-A3 and NY-ESO-1 between 5–20 fold in HEC1A cells and between 2–4 fold in ECC1 cells. In most cases the up-regulation could be confirmed by Western blot analysis using specific antibodies (Figure 7C).

### L1CAM is not expressed in human testis tissue

**Figure 8:**
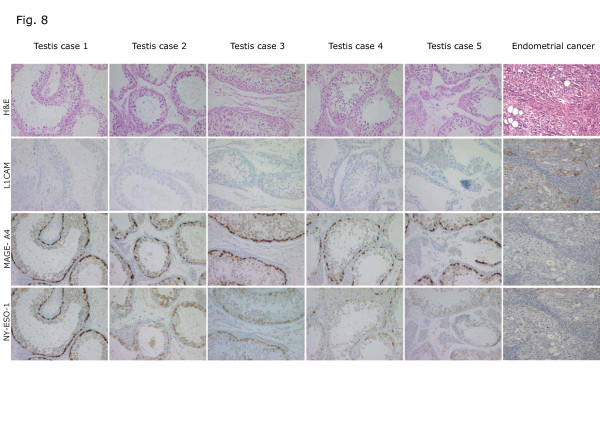
**IHC analysis of testis and EC tissues.** Expression of NY-ESO-1 and MAGE-A4 but absence of L1CAM in normal human testis tissue. Conversely, L1CAM is expressed in type II EC but NY-ESO-1 and MAGE-A4 are undetectable. Note that a representative case of n = 5 is shown. Sequential tissue sections were analysed by IHC.

It is known that CT-X antigens are expressed in human testis tissues. To further identify differences between L1CAM and CT-X antigens, we compared the expression of L1CAM, NY-ESO-1 and MAGE-A4 on a human testis tissue microarray using IHC staining. As shown in Figure 8, MAGE-A4 and NY-ESO-1 immunoreactivities were clearly detected but L1CAM staining was not. In contrast, when tested on EC tissues (n = 5), L1CAM was present but NY-ESO-1 and MAGE-A4 were not detected (Figure 8). These findings further support a different regulation of L1CAM and CT-X-antigens.

## Conclusions

Alterations in DNA methylation pattern which often occur during the pathogenesis of human tumours. Although DNA hypermethylation and the silencing of tumor suppressor genes has been the focus of such studies, a recent study in prostate cancer has shown that DNA hypomethylation can occur in distinct pattern due to longe-range epigenetic remodelling [12]. 35 activated domains harbouring cancer-related genes were identified present on nearly all chromosomes among them region Xq28 on the X chromosome [12]. As L1CAM and CT-X antigens are often expressed in tumors and are located in close vicinity on the X-chromosome it was of interest to investigate whether the regulation of these genes has similarities. Besides the methylation status of the respective promoter region, the configuration of the chromatin is also important. The chromatin can be modified by either histone acetyltransferases or HDACs, which are involved in post-transcriptional modification of histone proteins, resulting in chromatin remodelling [24].

Here we observed that L1CAM and CT-X antigens NY-ESO-1 and MAGE-A3/4 are equally sensitive to DNA methylation changes but differ in response to TSA induced regulation. CT-X antigens are a group of proteins that appear to be expressed only in germ cells, trophoblasts and various tumour types such as in carcinomas of bladder, lung, ovary and liver [25]. Many CT-genes have been identified so far, and they can be generally grouped into those, encoded on the X-chromosome (CT-X antigens) and those not encoded on the X-chromosome (non-X CT antigens) [25]. Frequently, tumours tend to co-express several CT-X genes [26]. In human tumours the aberrant expression of the CT genes which are normally epigenetically silenced during vertebrate development [27] are up-regulated by alteration in the genetic imprinting of the X-chromosomal regions [24]. Epigenetic mechanisms, i.e. an increased histone acetylation and a reduced DNA-methylation are involved in the aberrant activation of CT genes [24].

We found that in L1CAM high expressing EC cell lines the promoter 1 was hypomethylated whereas in low/negative cells this was not. Hypomethylation in the L1CAM promoter could influence the binding of transcription factors such as β-catenin/TCF-LEF and SLUG that are known to be involved in the regulation of L1CAM expression [14,22,23].

In contrast to the EC cell lines, a clear-cut difference in L1CAM promoter methylation of *ex vivo* tumor tissues was not found. Instead, we observed a high inter-individual variability of promoter methylation. In areas positive or negative for L1CAM within the same tumor no consistent differences were observed. Only in 3 out of 10 paired tumor samples from various EC types a tendency for hypomethylation in L1CAM positive tumor areas was noted. These findings contrast to the report by Kato et al. [11]. The authors analysed colorectal carcinoma cell lines and tumor tissues and found a good correlation between L1CAM immunoreactivity and methylation status [11]. It should be noted that the authors did not compare L1CAM positive and negative parts of the same tumor. Thus, in part the different findings could reflect differences in the study design and techniques employed. Another possibility is that additional mechanisms of regulation are involved in tumor tissues and that DNA methylation is not a critical factor for dynamic expression changes of L1CAM in the tumor microenvironment.

Finally, in contrast to the CT-X antigens NY-ESO-1 and MAGEA, there was no L1CAM expression detected in human testis tissue. The methylation status of the L1CAM promoter in testis tissue remains to be elucidated. These differences in regulation and expression in tumors suggest that L1CAM is most likely not a CT-X related antigen.

## Abbreviations

5′-Azacytidine: 5-AzaC; CT-X: Cancer testis antigens on the X chromosome; EC: Endometrial carcinoma; IHC: Immunohistochemistry; HDAC: Histone deacetylase; L1CAM: L1 cell adhesion molecule; mAb: Monoclonal antibody; TSA: Trichostatin A; VA: Valproic acid

## Competing interests

The authors declare no competing interests.

## Authors’ contributions

US designed and performed experiments shown in Figures 1, 2, 3, 4, 5, 6 and participated in writing the manuscript. US and HF designed experiments shown in Figures 4, 5 and Table 1, US carried out the sequence analysis. VT, PKB and EMH carried out the immunohistochemical analysis. MP and AZ participated in the experimental design and in writing and critically reviewing the manuscript. PA supervised the study and the experimental design and wrote the manuscript. All authors read and approved the final manuscript.

## Pre-publication history

The pre-publication history for this paper can be accessed here:

http://www.biomedcentral.com/1471-2407/13/156/prepub
