# Correction to: Epigenetic regulation of L1CAM in endometrial carcinoma: comparison to cancer–testis (CT-X) antigens

**DOI:** 10.1186/s12885-018-4928-y

**Published:** 2018-10-29

**Authors:** Uwe Schirmer, Heidi Fiegl, Marco Pfeifer, Alain G. Zeimet, Elisabeth Müller-Holzner, Peter K. Bode, Verena Tischler, Peter Altevogt

**Affiliations:** 1 Department of Translational Immunology, German Cancer Research Center, D015, D 69120 Heidelberg, Germany; 2 Department of Gynecology and Obstetrics, Medical University of Innsbruck, A 6020 Innsbruck, Austria; 3 Institute of Surgical Pathology, University Hospital Zürich, Zürich, Switzerland

## Correction

Following publication of the original article [1], we have been alerted to errors in Figs. 2 and 8. In Fig. 2b, the GAPDH loading control for Hec1A cells is shown twice in error (in Fig. 2b and Fig. 2c). In Fig. 8, in testis case 1 (first column) the MAGE-A4 staining panel was repeated and also appears as the NY-ESO-1 staining panel in error. The corrected versions of Fig. 2 and Fig. 8 are shown below. We apologize for this inconvenience.

**Fig. 2:**
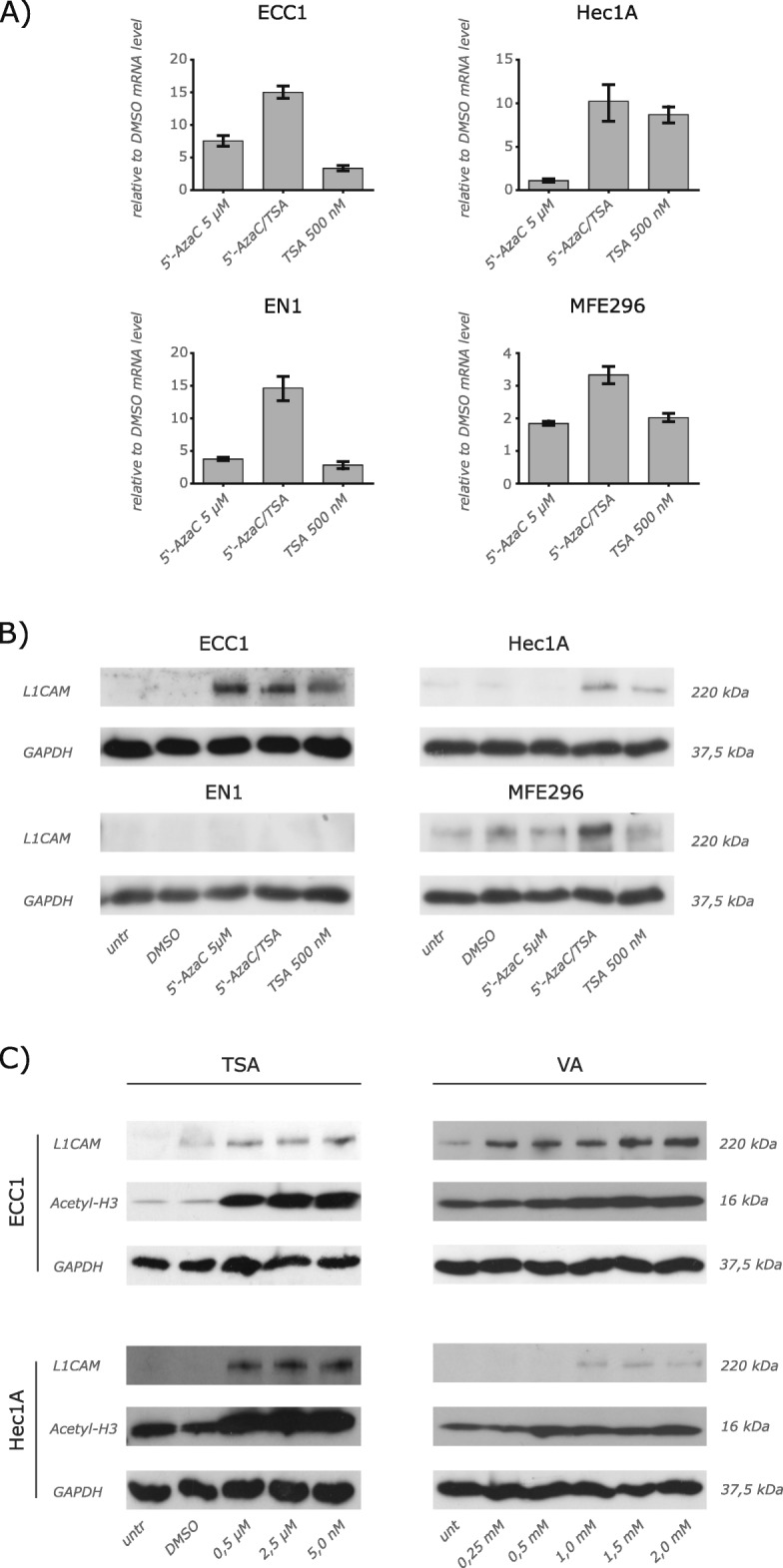
Regulation of L1CAM expression by epigenetic mechanisms. (**a**) RT-PCR analysis of cells treated for 5 days with the indicated concentration of 5-AzaC, TSA or both compounds. DMSO was used as a mock control. Cells were lysed and mRNA was isolated and transcribed into cDNA. β-actin served as internal standard. (**b**) Cells were treated as described above and cell lysates were prepared for Western blot analysis. MAb L1-11A was used as a primary antibody followed by peroxidase conjugated Goat anti mouse IgG and ECL detection. (**c**) TSA and VA up-regulate L1CAM expression. Cells were treated and analyzed as described in (**b**)

**Fig. 8:**
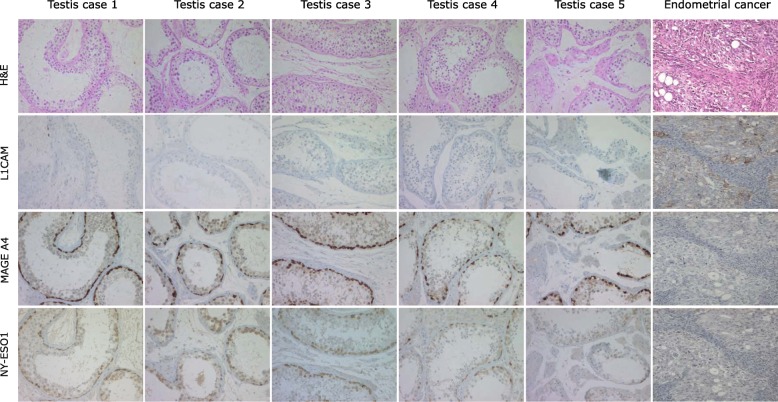
IHC analysis of testis and EC tissues. Expression of NY-ESO-1 and MAGE-A4 but absence of L1CAM in normal human testis tissue. Conversely, L1CAM is expressed in type II EC but NY-ESO-1 and MAGE-A4 are undetectable. Note that a representative case of *n* = 5 is shown. Sequential tissue sections were analysed by IHC
